# The Influence of CFRP Sheets on the Load-Bearing Capacity of the Glued Laminated Timber Beams under Bending Test

**DOI:** 10.3390/ma14144019

**Published:** 2021-07-18

**Authors:** Klaudia Śliwa-Wieczorek, Krzysztof Adam Ostrowski, Justyna Jaskowska-Lemańska, Anna Karolak

**Affiliations:** 1 Faculty of Civil Engineering, Cracow University of Technology, 24 Warszawska Str., 31-155 Cracow, Poland; klaudia.sliwa-wieczorek@pk.edu.pl; 2 Department of Geomechanics, Civil Engineering and Geotechnics, AGH University of Science and Technology, Al. Mickiewicza 30, 30-059 Cracow, Poland; lemanska@agh.edu.pl; 3 Faculty of Civil Engineering, Wroclaw University of Science and Technology, Wybrzeze Wyspianskiego 27, 50-370 Wroclaw, Poland; anna.karolak@pwr.edu.pl

**Keywords:** glued laminated timber, CFRP, load-bearing capacity, force–displacement relationship, bending test, CT analysis

## Abstract

Composite materials are increasingly used to strengthen existing structures or new load-bearing elements, also made of timber. In this paper, the effect of the number of layers of Carbon Fiber Reinforced Polymer (CFRP) on the load-bearing capacity and stiffness of Glued Laminated Timber beams was determined. Experimental research was performed on 32 elements—a series of eight unreinforced beams, and three series of eight reinforced beams: with one, three and five layers of laminate each. The beams with a cross-section of 38 mm × 80 mm and a length of 750 mm were subjected to the four-point bending test according to standard procedure. For each series, destructive force, deflection, mode of failure, and equivalent stiffness were determined. In addition, for the selected samples, X-ray computed tomography was performed before and after their destruction to define the quality of the interface between wood and composite. The results of the conducted tests and analyses showed that there was no clear relationship between the number of reinforcement layers and the load-bearing capacity of the beams and their stiffness. Unreinforced beams failed due to tension, while reinforced CFRP beams failed due to shear. Despite this, a higher energy of failure of composite-reinforced elements was demonstrated in relation to the reference beams.

## 1. Literature Survey

Wood is a widely used natural construction material that has excellent properties when compared to other construction materials. The advantages of wooden structures include their light weight, relatively high strength, ease of processing, good fire resistance, high aesthetics, and competitive costs. Glulam is used as a material that has improved properties when compared to solid wood, which can mainly be applied in bar structures with large spans—typically up to 50 m. Such large spans are obtained by using finger joints along the entire length of the elements. As wood is an organic material, it may deteriorate over time and require strengthening, especially when it is not properly maintained [1]. The typical causes of degradation include infestation, fungi (biological corrosion), chemical corrosion, and also the influence of atmospheric conditions (temperature gradients and changes in humidity) [2]. In addition to the destructive impact of the environment, it may be necessary to increase operational loads as a result of the following: changes in the use of a structure, its repair, the improvement of its static work due to design or manufacturing errors, the reduction in deformations, or the occurrence of mechanical damage. Traditional methods of repairing wooden structures used to involve the use of wood (e.g., overlays, which were most often fixed with mechanical fasteners), or steel sections. Along with the development of the gluing technique (especially in the field of epoxy adhesives), reinforcements started to be made by gluing steel bars and sheets (flat bars) in order to improve the behavior of a structure [3,4,5]. One of the commonly used traditional methods of repairing or strengthening structural elements involves the strengthening of their external surfaces or cross-section (along the entire length of the element, only in weakened zones, or by filling in missing parts—most often at the ends of the beams). The reinforcement may be in the form of steel plates and bars, and sometimes also additional reinforcing elements, such as, e.g., steel cords (described in [6]). The disadvantages of traditional reinforcements include their relatively high weight, difficulties associated with their implementation (e.g., too small an area for the arrangement of connectors, stress concentration), the small improvement of load-bearing capacity, and their susceptibility to corrosion.

Currently, Fiber-Reinforced Polymer composites (FRP) are most often used to strengthen wooden, concrete and masonry structures. Fiber Reinforced Polymer (FRP) is a composite material made of a polymer matrix reinforced with fibers. Due to the numerous advantages mentioned below, the use of FRP materials is becoming more and more common, also in the case of wooden structures. Generally, this technique is mainly used to strengthen existing elements. However, newly designed hybrid structures can also be found, and they are becoming increasingly popular. Advantageous mechanical parameters, a favorable ratio of strength and stiffness to weight, resistance to aggressive environments (no corrosion), good fatigue properties, and low life cycle costs are the main advantages of FRP [7,8,9,10,11]. Moreover, FRP materials can be easily adjusted to the curvature of reinforced elements, and also can be quickly assembled [12]. In addition, the thermal conductivity of composites is much lower than that of, e.g., steel, and therefore a reinforced structure may be more resistant to fire [13]. A comparison of reinforcing inserts in the form of steel sheets and CFRP strips, which are used to strengthen solid and glued timber beams, is presented in [14,15]. FRP materials and wood can cooperate better with each other because they have similar thermal expansion. What is more, as some examples show, according to [16], the internal GFRP reinforcement, besides improving load-bearing capacity in bending, may also decrease the influence of materials’ natural flaws on mechanical properties and positively affect reached characteristic values and lower variability of the results.

A description of the technique of strengthening wooden elements with the use of the external adhesion of pre-stressed FRP sheets in their tensile zones, as well as a methodology for selecting the dimensions of a composite material, can be found in [17]. The most commonly used composite is CFRP (Carbon Fiber Reinforced Plastics) in the form of tapes and mats. Experimental tests and modelling of glued timber beams reinforced with FRP including CFRP are described in detail, among others, in papers [18,19,20,21,22,23]. In addition, a model approach to predict the behavior of wooden beams reinforced with CFRP was presented. Three-dimensional models of finite element analysis (FEM) were formulated based on the orthotropic constitutive characteristics of the types of timber [11]. Moreover, models of the finite elements, which are calibrated based on experimental data, can also be found [12]. One of the first descriptions of the reinforcement of wooden beams was presented by Bulleit [24]. He evaluated the results of testing laminated beams with fiberglass started by researchers in the 1960s until the 1980s. He pointed out that this is a favorable future direction.

It is also worth mentioning that there is a possibility of using FRP, including CFRP, to strengthen the structural elements of monumental or historical buildings—even those characterized by a very poor technical condition, incl. [13,19,25]. The main advantage when strengthening monuments is the possibility of obtaining a sufficiently high strength with a low weight and mass of the used reinforcement. In addition, the reinforcing material can be located in such a way that the original appearance of the element is restored, which is extremely important in the case of historic buildings, and acceptable with regard to conservation doctrine [26]. Usually, in the case of historic buildings, it is not possible to use external reinforcement on the surface of ceiling beams. In the case of ceilings with rich decorations, e.g., carvings, bas-reliefs, or polychromes (with woodcarvings or polychromes), the preferred solution is to strengthen the wooden cross-section. Additionally, the reinforcement of the cross-section limits the possibility of delamination of the adhesive joint that strengthens the wood, and, as already mentioned, increases fire resistance [3]. This type of reinforcement was successfully used in the strengthening or conservation of a number of historical and monumental objects of high value, such as the wooden bridge near Sins in Switzerland from 1807 [27,28], the Italian palace Palazzo Nobili [29], or the Gothic church in the German town of Meier [30]. A very interesting Polish example is the historic telecommunication tower in Gliwice, which was erected in 1933 [31]. This structure was reinforced with CFRP tapes. The concept of strengthening the tower using metal bands was also considered. However, this solution, due to the violation of the aesthetics of the building, was not approved by the conservator of monuments. FRP materials used in the strengthening or repair of wooden elements are most often in the form of bars and strips, which are glued to the wood with a mixture of epoxy adhesives. FRP materials are not only used to strengthen wooden elements of a certain historical value, but can also be used to strengthen bent Glued Laminated Timber elements during their production [12,32,33].

In the process of wood strengthening, it is very important to properly execute the bond between the wood and the reinforcement kit (composite). Thus far, several studies have been performed to investigate the bond behavior of strengthened timber beams. For instance, in [34], the authors analyzed the bonding with regard to moisture effect and different types of epoxy adhesives applied. The study showed that the application of used adhesives is capable of resisting even severe hygrothermal stresses at the interface between wood and composite. What is more, the authors explored the bond integrity on the epoxy adhesive as well as the applied type of FRP. A similar issue was analyzed in [35]. In this case, the study also confirmed that epoxy adhesives have good resistance to different hydrothermal conditions. In turn, in [36,37], the authors presented a description of the bond behavior between wood and FRP tested by the use of pullout tests. According to the authors, the presented analytical models [37] may be used as reliable tools in the designing process of such connections.

**Figure 1:**
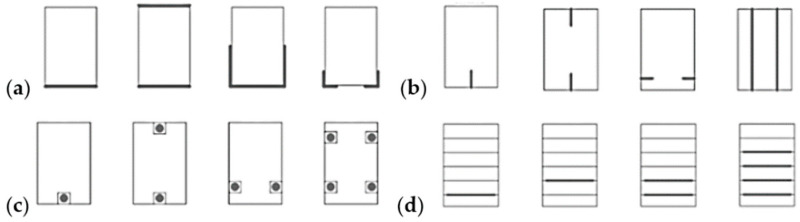
Methods of strengthening wooden beams with the use of CFRP strips according to [46]: (**a**) perimeter reinforcement, (**b**) internal reinforcement, (**c**) glued bars, (**d**) interlayer reinforcement for Glued Laminated Timber beams.

One of the main causes of the weakening of the level of reinforcement is the quality of the interfacial bond between wood and FRP [38,39,40]. As described in [41,42], one of the frequent failure mechanisms of reinforced beams is the premature detachment of FRP tapes before the maximum destructive deformations are obtained. As a consequence, such a failure means that the reinforced structure will not obtain the desired load-bearing capacity and plasticity. In 1999, Dagher [43] observed that is possible to increase bending strength by up to 100% by strengthening beams on only their bottom surface. It is also common that an increase in strength depends on the reinforcement that was used on the analyzed cross-section of the structure. However, this increase is not proportional to the percentage of the used reinforcement. This is due to the fact that damage occurs at the laminate interface before the reinforcement (which is exhausted) causes shear cracks. It has been proven that the bending failure of the beams changes depending on the properties of the reinforcement that is used [44]. Another important factor concerns the properties of the beams to be reinforced, e.g., the occurrence of cracks, natural defects, or wood defects, which may cause premature failure of the element. The influence of wood defects on the strength of elements and the methods of protecting the side surfaces of beams against the exceeding of shear stresses were analyzed and investigated [45]. Figure 1 shows the typical methods of reinforcing sawn timber with CFRP strips.

There are many factors influencing the degree of increasing the strength parameters of reinforced beams, such as the type of the reinforced element and FRP material, the localization of reinforcement (as shown in Figure 1), the quality of cooperation between the FRP and the wood (joint integrity), the degree of reinforcement, etc. Therefore, the results presented by the authors of many papers differ depending on the above-mentioned aspects.

The obtained strengthening ranged from 20 to 40% in the case of unidirectional reinforcement with the use of carbon fiber composites when compared to unreinforced reference beams [16]. De la Rosa Garcia et al. conducted the analysis of the load-bearing capacity and flexural stiffness of timber beams reinforced with carbon and basalt composite in [47]. They presented a significant increase in the stiffness and flexural capacity of reinforced beams in comparison to the beams without reinforcement. In the paper presented in 2005 by Schober [44], an increase in bending stiffness of 5.86% in relation to unreinforced beams was obtained. Basterra et al. [48] obtained an increase of 23% in load-bearing capacity and an increase of 12–15% in stiffness using internal GFRP reinforced for laminated duo beams of low-grade timber. Nowak et al. [19] in 2013 observed an increase in the load-bearing capacity of beams reinforced with CFRP tapes ranging from 21% up to 79%. The differences in the obtained values result from the use of different types of reinforcement, wood, etc. In turn, Borri et al. in [49], besides experimental research, presented analytical investigation on the behavior of a generic FRP-reinforced wood section, numerical analysis. They described both linear and non-linear analysis. The authors distinguished two different failure mechanisms. The first one is the possibility of attaining wood tension stress, and the other appears while reaching the limit compression stress. In the experimental part of research, the authors obtained the maximum load increase of about 40 up to 60% for the sheets, an increase of about 30 up to 50% for the bars, an increase in stiffness of about 20 up to 30% for the CFRP sheets and the maximum load and an increase in stiffness of about 20 up to 30% for the CFRP sheets depending on the type of used strengthening. In the numerical part of research, they conducted analysis based on non-linear wood properties, which are considered suitable for application in the design of FRP reinforcement of old, pre-existing wood beams under varying configurations of intervention layouts and materials. The results from this part are very similar with the error level 2–4%, which can be considered a satisfying result. Schober et al. [50] analyzed FRP reinforcement of timber structures and presented, i.a., possible failure modes that can occur in the structure and should be considered. The following modes were distinguished: mode 1—failure of the timber in tension while in compression the response is linear elastic, and mode 2—failure of the timber in tension after the onset of compressive yielding. 

## 2. Research Significance

Currently, Glued Laminated Timber (GLT) is one of the most popular structural building materials in wooden constructions. It is a material that has more favorable stress–strain characteristics when compared to solid wooden elements. Along with the progress of technology in the construction industry, there are situations in which a GLT material requires the improvement of its properties by strengthening. This may be because there is a need to improve the functional properties due to the change in the use of a facility, a reduction in deflections, or an increase in the stiffness of the elements. Therefore, it was proposed to strengthen GLT with the use of the most commonly used fibers in the construction industry (from the group of FRP materials)—carbon fibers. This article analyzes the effect of the number of applied layers on the value of the force that was transferred by the tested beams. Moreover, the integrity of the connection between the composite and the beam was verified using X-ray computed tomography. Attention was paid to the effectiveness of the reinforcement, the course of damage, and the impact of the wood defects that possibly contribute to failure.

## 3. Materials and Methods

### 3.1. Materials

Sixteen beams with a cross-section of 38 mm × 80 mm and a length of 750 mm were tested. Four groups of samples were distinguished: unreinforced beams, and beams reinforced with 1, 3 and 5 CFRP layers. Four elements were tested in each group. They were marked as follows: Beam X/Y, where X is the number of CFRP reinforcement layers and Y is the number of the sample in a given series.

The beams were cut from glued laminated spruce wood, which had the GL28h class that was declared by the manufacturer in accordance with the PN-EN 14080: 2013-07 standard [51]. The characteristic mechanical properties of wood in the case of the GL28h class are as follows: bending strength f_m,g,k_ = 28 MPa, tensile strength f_t,0,g,k_ = 22.3 MPa, compressive strength f_c,0,g,k_ = 28 MPa, modulus of elasticity E_0,g,mean_ = 12.6 GPa. SikaWrap 300C carbon fiber mats (Sika Group, Baar, Switzerland) were used as reinforcement, which had the following properties declared by the manufacturer: f_t,cf_ = 4000 MPa, E_f_ = 230 GPa and δ_cf_ = 1.7%. The grammage of unidirectional reinforcement was equal to 304 g/m^2^ ± 10 g/m^2^ [52]. The mechanical parameters for the carbon fibers were determined in accordance with the PN-EN ISO 10618: 2006 standard [53]. In the lamination process, Sikadur 300 epoxy resin (Sika Group, Baar, Switzerland) was used as a binder, with the following parameters declared by the manufacturer: F_t,er_ = 45 MPa, E_er_ = 3.5 GPa, and δ_er_ = 1.5%. The mechanical properties of the epoxy resin were determined on the basis of the PN-EN ISO 527 standard [54].

### 3.2. Lamination Process

**Figure 2:**
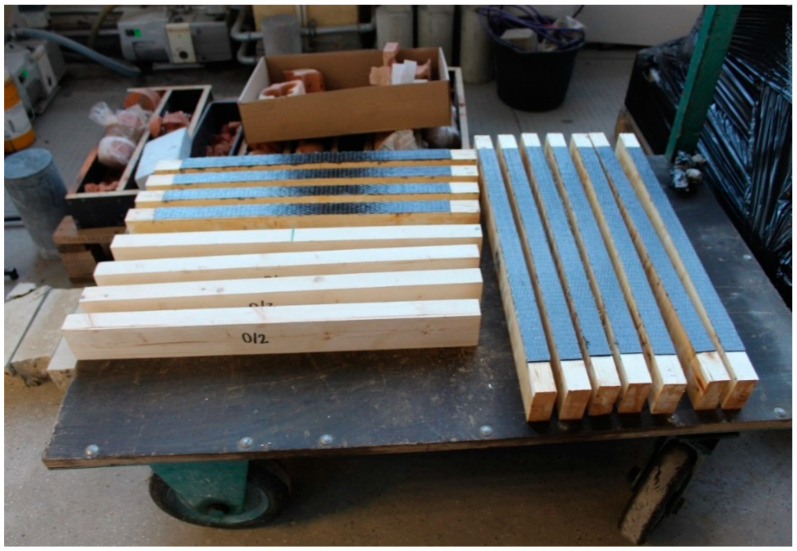
Beams prepared for destructive testing: unreinforced beams and beams reinforced using CFRP Glued Laminated Timber.

The effectiveness of strengthening structural elements also depends on the lamination process being performed correctly. The wooden beams were reinforced using the “dry lay-up process” method. The name of the “dry lay-up method” is related to the state of the FRPs at the time of their application in their final location. The epoxy resin applied in this method is used to strengthen the surface of the wooden element and to impregnate the FRP. In this process, in the first step, epoxy resin was applied to the wood element with a brush. Afterwards, the previously prepared reinforcement was placed on the element in the form of a suitably cut mat of carbon fibers, which was then laminated in the direction of the fibers with the use of a roller. In the case of making three and five layers of CFRP reinforcement, the common “wet on wet” method (which is recommended by the manufacturer and contractors) was used, i.e., applying a wet layer onto a wet one. The reinforcement of the beams for each series was made in the tensile zone using a 65 cm long mat. The width of FRP reinforcement was the same as beam width. The length of the reinforcement was selected in order to reflect the real conditions of the reinforcement in the existing structure, i.e., outside the support area. Figure 2 shows the unreinforced and CRFP-reinforced beams that were prepared for destructive testing.

### 3.3. Bending Test

**Figure 3:**
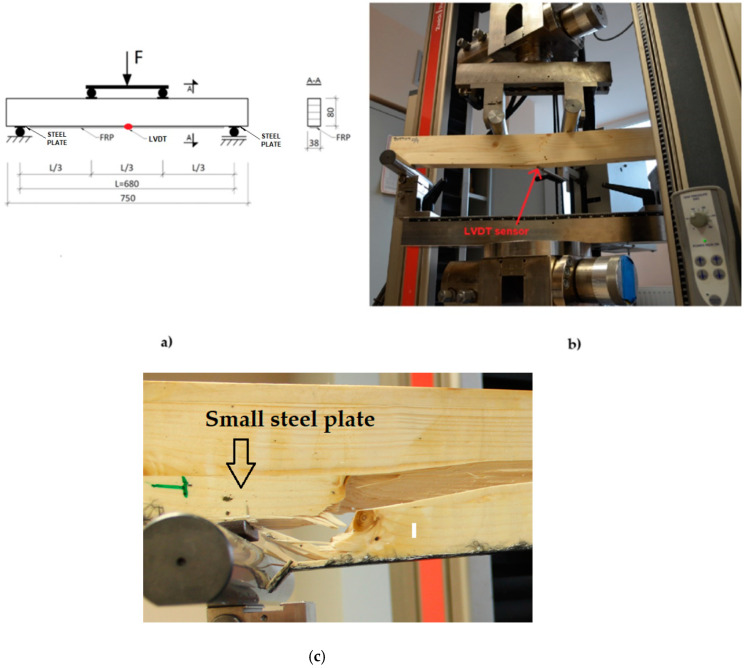
The test set-up: schematic view (**a**), view of the beam on the test stand (**b**) and view of the steel spacer on the support (**c**).

The experiment was carried out on beams subjected to the four-point bending test, as shown in Figure 3a. The increase in the breaking load was achieved by ensuring a constant increase in the displacement (deflection) of the tested elements, which was 2 mm/minute. The speed of the element displacement increase was selected so that the destruction of the beams took place within 300 ± 120 s, which is in accordance with the standard [55]. The test was carried out using the ZWICK 100 (Zwick Roell Group, Ulm, Germany) testing machine (Figure 3b). The span between the axes of the supports was 680 mm. The tests were conducted at a temperature of 23 °C ± 1 °C and a humidity of 60% ± 5%. Vertical displacements were measured using the LVDT sensor (Figure 3) in the middle of the beam span. Due to the difference in the deformability of glulam timber and steel supports, the authors want to alert readers that indentation of supports in the wood structure may occur. As during the experiment no additional LVDT sensors were available near the supports, in order to minimize errors in the measurement of the global displacement (avoiding a point indentation of the wood) on the rollers of the supports, in accordance with the provisions of PN-EN 408 additional spacers made of small steel plates were used (Figure 3c). Despite the use of the steel plates, some adaptation of the contact zone between different materials probably occurred in the initial part of the test because the load–displacement curves started to be concave up, before they take the concave down normal shape for almost the total length. In summary, the initial disturbances in the force–displacement diagrams are the result of fitting elements near the supports (between steel supports, steel plates and glulam beam) in the initial phase of the test.

**Figure 4:**
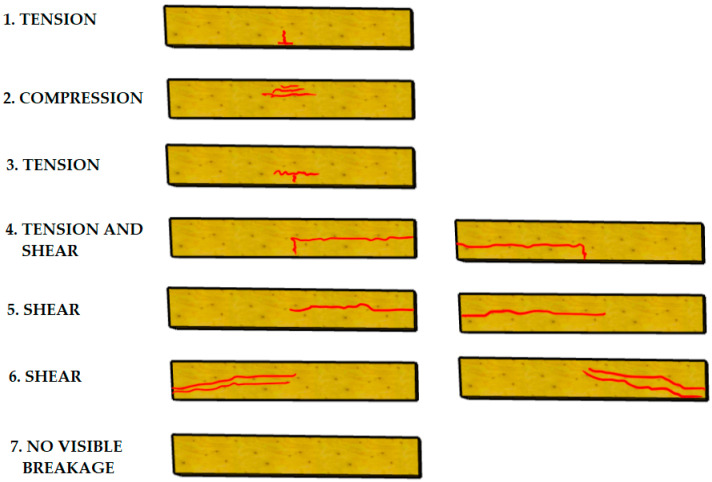
Classification of failure modes in the timber beams subjected to bending according to [56].

The load was applied until the beams were destroyed, and then force–displacement plots were made for each element. The failure of a sample was classified according to the seven types of failure considered in the De la Rosa et al. study [56], which are shown in Figure 4.

### 3.4. X-ray Computed Tomography Test

**Figure 5:**
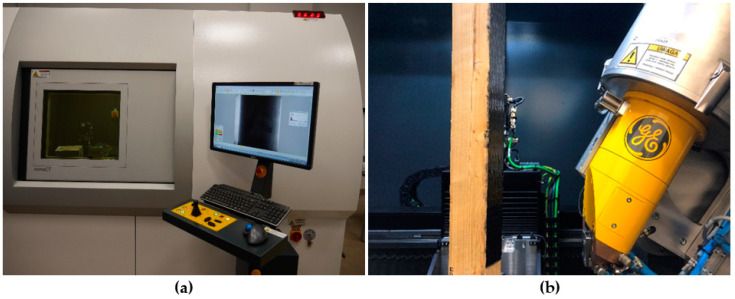
X-ray CT examinations: (**a**) the GE Phoenix v-tome-x m device during the test; (**b**) a sample prepared for tomographic examination mounted inside the device.

One representative sample from each group was subjected to X-ray computed tomography, which showed the structure of the wood and CFRP reinforcements. The tomography was performed after the application of the reinforcement, and also after the destructive tests with the use of the GE Phoenix v-tome-x m device (General Electric Sensing & Inspection Technologies GmbH, Pforzheim, Germany) which is shown in Figure 5. The test after the application of the CFRP reinforcement was performed in the middle of the span of the elements, which allowed the structure of the connection between the reinforcement and the beams to be visualized. In turn, post-failure tests were performed on the entire length of the elements, which enabled detection of critical sections with regard to failure.

The GE Phoenix v-tome-x m device with VG Studio Max software enables the internal structure of the tested element to be reconstructed and analyzed on the basis of a series of X-ray images taken during the 360 rotation of the tested element. Table 1 shows the basic test parameters and their ranges of values.

## 4. Results

### 4.1. Load-Bearing Capacity

The results of the research, with a comparative analysis, are presented below. The method of destroying the reference beams and the beams reinforced with one, three and five layers of CFRP is described. During the destructive tests, the destructive force achieved by individual test elements and also the vertical displacement in the middle of the span were recorded. Their values are presented with the equivalent stiffness parameter EI in Table 2. The equivalent stiffness for individual beams was determined as the average value by transforming Formula (1) into the deflection of a single-span beam loaded for the displacement range from 2 to 6 mm (with a reading of the force increment every 1 mm). The proposed range for which the equivalent stiffness was determined in the deflection range of 2–6 mm and according to the authors allows us to obtain a value at a satisfactory level of accuracy—a slight indentation in the glulam beam in the area of supports (for the proposed range of vertical displacement) will be negligible. In this case, the force increment to the displacement was similar to linear.

(1) $${\mathrm{EI}}_{\mathrm{mean}\left(u=2–6\;\mathrm{mm}\right)}=\frac{1}{n}\cdot \overset{n}{\underset{i=1}{\sum }}\frac{23\cdot F\cdot l^{3}}{648\cdot u}$$

(2) $${\mathrm{EI}}_{X/Y}=\frac{1}{k}\cdot \overset{k}{\underset{j=1}{\sum }}{\mathrm{EI}}_{\mathrm{mean}\left(u=2–6\;\mathrm{mm}\right)}$$

where: 

F—force (N), l—span of an element (m), u—deflection (m), n—number of records of force F within the displacement range of 2–6 mm (increment of displacement = 1 mm), k—number of samples in a given series.

Table 2 shows the values of: the destructive force “F”, vertical displacement “u” for the maximum force and the value of the stiffness “EI” for each series of specimens.

**Figure 6:**
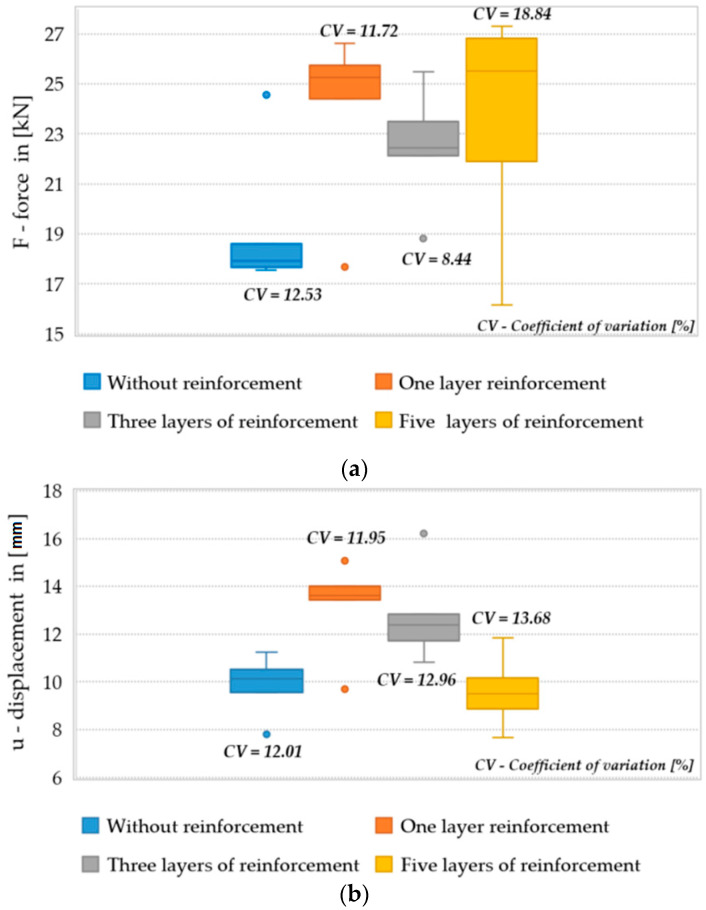

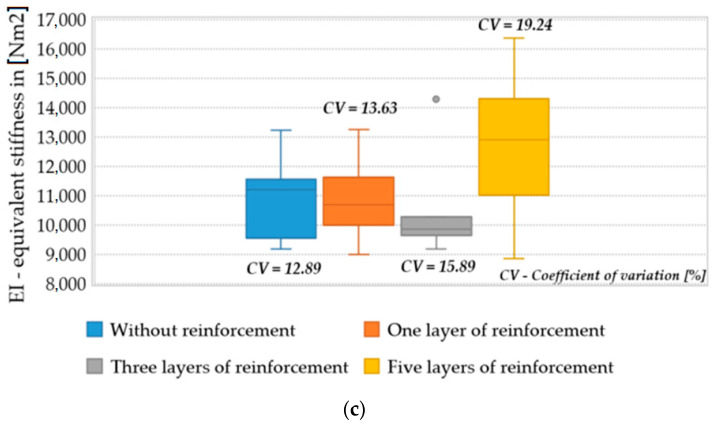
Box and whisker plots for (**a**) maximum destructive force; (**b**) displacements under destructive force; (**c**) equivalent stiffness for the range 2–6 mm.

The box and whisker plots for the trials are presented below in Figure 6. The charts also contain information on the values of the coefficient of variation.

As can be seen from the diagrams for beams without reinforcement and with one and three layers of reinforcement, atypical values are observed, differing from the others (marked in the diagram as a circle). These are the values of the outlier measurement (1.5 to 3 quarter range values). As these values distort the mean values for the performed samples, they were rejected in further analyses. For reference beams and beams with one and three reinforcement layers, one measurement was rejected. Table 3 shows the mean value of the destructive force and bending moment.

In the case of the reference beams, the mean value of the destructive force was equal to 17.99 kN (for seven specimens, one extreme result was rejected), and had a standard deviation of 0.45 kN. The mean value of the vertical displacement, measured in the middle of the span of the beams, was equal to 9.76 mm. The mean value of the equivalent bending stiffness for the verified interval was equal to 10,540 Nm^2^, with the standard deviation being equal to 1114 Nm^2^.

For the beams reinforced with one layer of CFRP, a clear increase in the mean value of the destructive force was observed. The value of this force was equal to 25.38 kN (for seven specimens, one extreme result was rejected), and the standard deviation was 0.96 kN. The increase in relation to the average load-bearing capacity of the beams without the reinforcement was 41.07%, which proves the significant positive impact and effectiveness of the used reinforcement. For the beams of this series, the mean value of vertical displacement, measured in the middle of the beam’s span, was equal to 13.92 mm. It was greater than the values obtained by the beams without the reinforcement, but was recorded with a greater force. The mean value of the equivalent bending stiffness was equal to 11,177 Nm^2^, which is more than that of the unreinforced beams.

The beams reinforced with three layers of CFRP also achieved a higher destructive force than the reference beams equal to 23.11 kN (for seven specimens, one extreme result was rejected), and had a standard deviation of 1.24 kN. The recorded increase in the load-bearing capacity was approx. 28.46%. The series with three layers was characterized by the highest repeatability of the obtained results for the force F (the coefficient of variation was 5.24% for seven specimens). The average vertical displacement for the beams of this series was 12.76 mm, and was greater than in the case of the beams without the reinforcement. The average value of the equivalent bending stiffness for the beams of this series was 10,452.40 Nm^2^. For the series with three layers of the reinforcement, no increase in stiffness was obtained in the analyzed range, and the obtained value was the lowest value recorded during the experiment. In turn, for the beams reinforced with five CFRP layers, the average value of the destructive force was equal to 23.62 kN (for eight specimens), and the standard deviation was 4.45 kN. The increase in the load-bearing capacity in relation to the unreinforced beams was 31.29%. The average vertical displacement for this series was 9.60 mm, but it is worth noting that the coefficient of variation for this series was the largest—over 13.60%. The obtained scattering of results may, however, be related to the heterogeneity of the material (wood) and its nature. Therefore, it is difficult to conclude that the use of five layers of CFRP is ineffective. This is especially due to the fact that the beams from this series were destroyed at the place of inclusions in the wood (knots in the support zones), and thus the obtained values were lower than expected.

**Figure 7:**
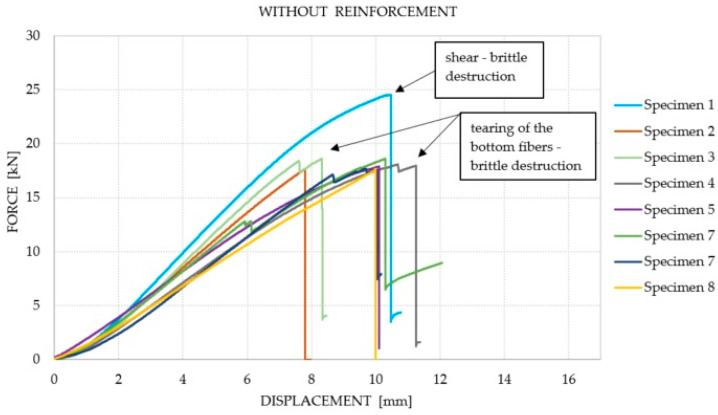
Force–displacement chart for the beams without reinforcement.

**Figure 8:**
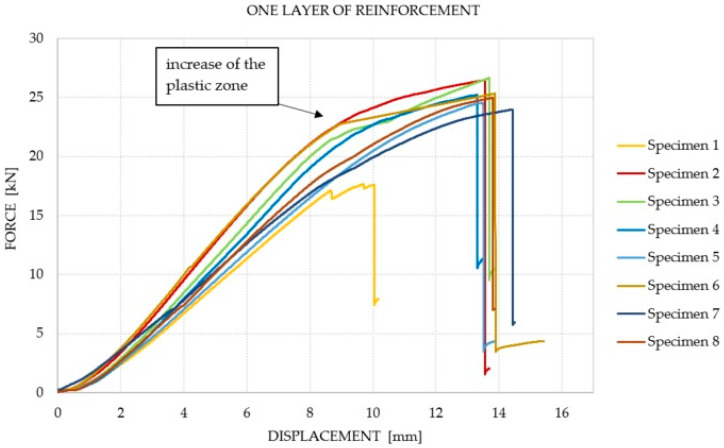
Force–displacement chart for the beams with one layer of reinforcement.

**Figure 9:**
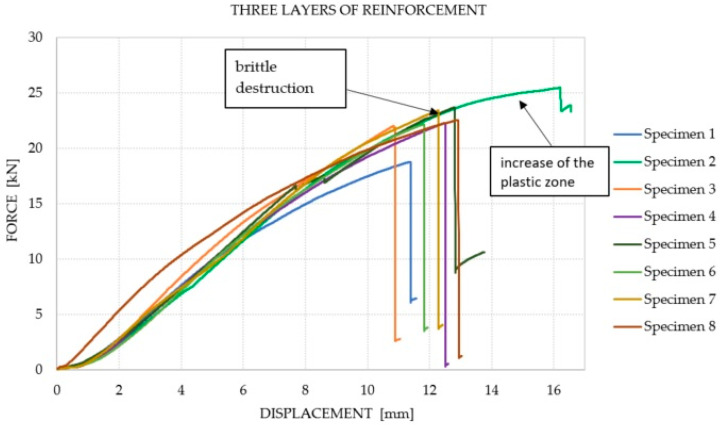
Force–displacement chart for the beams with three layers of reinforcement.

**Figure 10:**
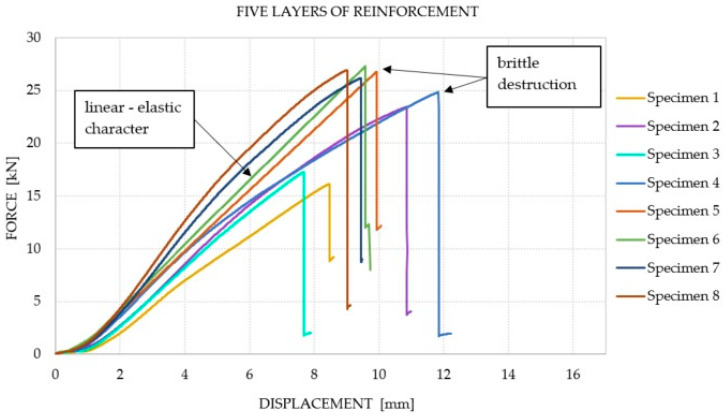
Force–displacement chart for the beams with five layers of reinforcement.

It was observed that the number of reinforcement layers did not affect the increase in the average equivalent stiffness within the considered range (between 2 and 6 mm) of displacements, in which the course of the chart was the closest to linear. It was shown that a greater amount of reinforcement does not translate into an increase in the bending capacity of the elements. The dominant factor that influenced the failure of the Glued Laminated Timber beams was the strength of the wood and the number of defects and their location. The course of the force–displacement curve for each of the analyzed groups is presented in Figure 7, Figure 8, Figure 9 and Figure 10. During the bending test, wood is characterized by elastic–plastic behavior, and the destruction of this material is signaled by the change in the nature of its behavior from linear-elastic to non-linear-elastic. Failure most often occurs by tearing the bottom fibers (the first cracks in the fibers are visible for series 3 and 4—a characteristic abrupt loss of stiffness in the diagram), and is of a brittle nature, as shown in Figure 9 and Figure 10. Due to the use of reinforcement made of composites, it is visible that the tensile zone of the beams is protected (the nature of the damage changes, the beams are not damaged by the breaking of the fibers in the tensile zone). Reinforcement, especially that of one layer, increased the scope of the plastic zone, and also translated into greater deformability where the failure occurred. All the beams with one layer of reinforcement (excluding series 1) achieved a displacement greater than 13.29 mm, which is a higher value than the average deflection for each series. On the basis of the chart and the obtained results, it can be concluded, in the case of the performed test, that this was the most optimal out of all the considered reinforcements. For the beams reinforced with three layers, an intermediate effect was obtained, i.e., deformability was increased when compared to the reference series; however, the increase within the range of the plastic zone was not as large as for the beams with one layer of reinforcement. The implementation of five reinforcement layers changed the nature of the material’s behavior into linear-elastic (the plastic zone was strongly limited). The beams were destroyed in a brittle manner, and the force–displacement charts were similar to those of the reference beams.

The course of the failure of the beams was determined on the basis of the De la Rosa [56] classification, which is presented in Table 4. It was shown that the number of CFRP layers had no effect on the failure mode of the tested elements.

**Figure 11:**
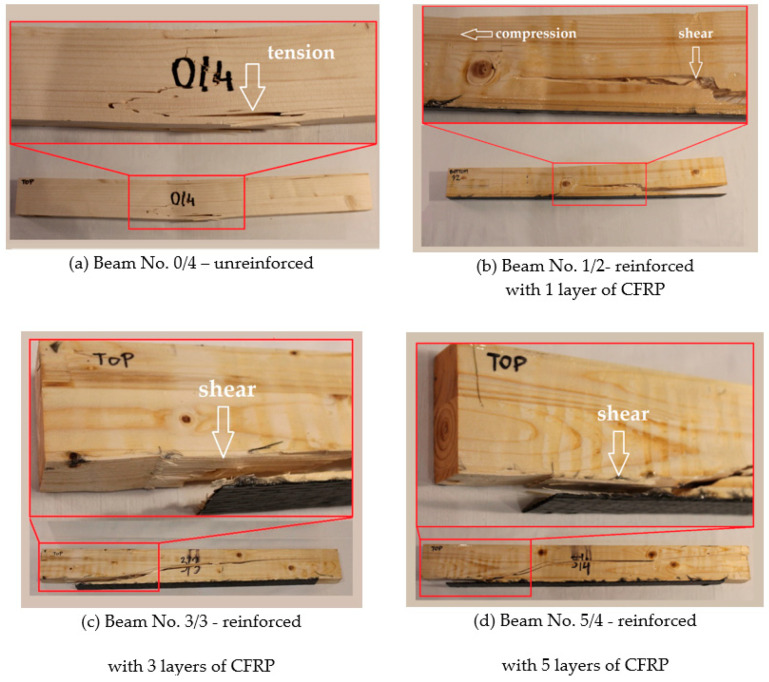
Representative examples of failure for each of the analyzed groups of samples. Beam No. 0/4 (**a**), Beam No. 1/2 (**b**), Beam No. 3/3 (**c**), and Beam No. 5/4 (**d**).

Figure 11a–d show representative examples of the samples’ failure in the case of the individual groups. There was no breakage of the CFRP reinforcement fibers in any of the observed cases. It was shown in the case of the Glued Laminated Timber beams that knots in the wood cause them to crack. This significantly affects the beam’s stiffness during bending, and also determines the damage. It was proven that the location of knots in the tensile zone of the beam (which is important in the case of bending) reduces its strength, regardless of whether CFRP reinforcement is used.

### 4.2. X-ray Computed Tomography Analysis

**Figure 12:**
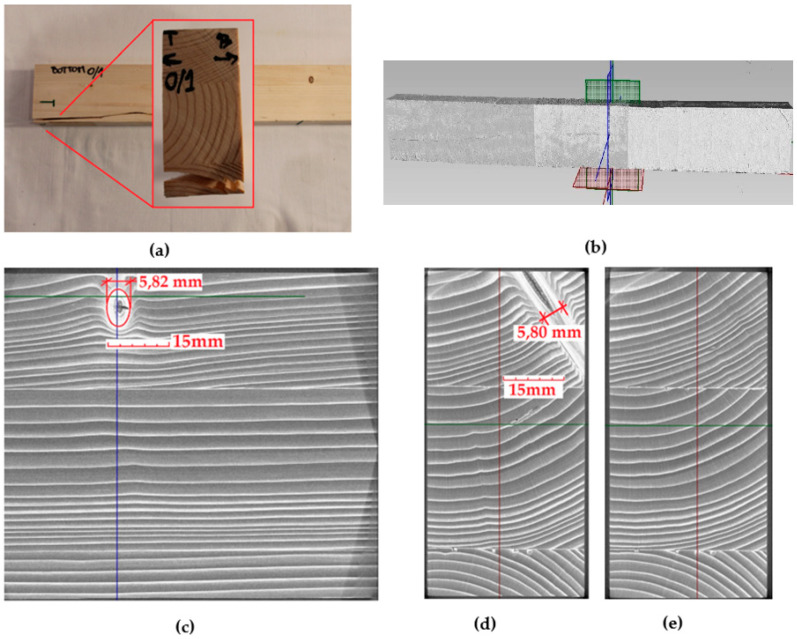
Part of reference Beam No. 0/1—condition after failure: (**a**) view of the failure, (**b**) view of the scanned beam, (**c**) longitudinal cross-section in the middle of the span, (**d**) cross-section in the place of the knot, (**e**) transverse cross-section in the middle of the span.

Due to the use of X-ray computed tomography analysis, the number of wood defects and the quality of the connection between the CFRP and the timber substrate were determined. In the case of the reference beams, the highest value of destructive force for this series was transferred by beam 0/1 (value 24.55 kN, which is 24% more than the average value of the destructive force for the entire series). Moreover, it was the only beam in the series that was not damaged by the tension of the bottom fibers. It was shown that this is undoubtedly related to the fact that it had the smallest number of defects (only one knot with a diameter of more than 5 mm was found in the compressed zone of the beam), as can be seen in Figure 12. As can be noted in Figure 12c–e, the wood fibers were not disturbed in the tensile zone, and the occurring knot did not affect the nature of the failure.

**Figure 13:**
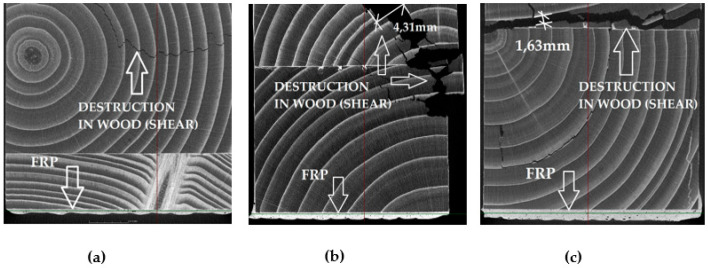
Cross-sections in the middle of the span for selected beams—state after the destruction: (**a**) Beam No. 1/4, (**b**) Beam No. 3/4, (**c**) Beam No. 5/2.

The CT examination before the application of the load, and also after the failure of the beams, showed a very good integrity between the beams and the CFRP reinforcement, as well as no damage to the reinforcement. The mid-span cross-sections are shown for selected beams from each series in Figure 13. There was no detachment of the CFRP reinforcement from the timber substrate in any analyzed case, which proves that the lamination process was correct, as well as that the reinforcement was continuous in all directions. In the case of each beam, the destruction took place in the wood, most often due to shearing in the bottom part of the cross-section in the support zone, and then ran to the center of the cross-section. The width of the split in the middle of the cross-section was much greater for the beams reinforced with three and five layers when compared to those with one layer of reinforcement.

## 5. Conclusions

This paper describes the influence of the number of Carbon Fiber Reinforced Polymer layers on the load-bearing capacity of Glued Laminated Timber beams during the bending test. The following conclusions can be stated from the work presented in the article:

1. In the Glued Laminated Timber beams subjected to bending, the dominant failure was the failure caused by the exceeding of the limit tensile stresses, and the crack occurred on the bottom surface of the beam. The most common type of failure was the breaking of the bottom fibers in the tensile zone, which was rarely accompanied by shear.
2. CFRP-reinforced beams exhibit linear elasticity until the moment of failure. Strengthening the bottom surface of a beam by gluing a composite allows its ability to absorb tensile stresses to be extended, which leads to an increase in the load-bearing capacity and ductility of the beam. During the conducted experiment, there were no breaks in the reinforcement fibers.
3. The computed tomography examination before the application of the load, and also after the destruction of the beams, enabled the very good integrity between the beams and CFRP to be confirmed—regardless of the number of reinforcement layers, the laminate did not detach.
4. It was noticed that the number of CFRP laminate layers has an influence on the mechanism of failure. For the beams reinforced with three and five layers, the dominant failure mechanism began with a crack at the connection of the wood and the adhesive layer in the support zone. This is related to the phenomenon of the concentration of interfacial shear and pull-off stresses. In practice, this phenomenon may have a negative impact on the final strength of reinforced elements.

## Figures and Tables

**Table 1:** Parameters of the X-ray CT examination.

Parameter	Value
Source voltage	60 kV
Source current	900 μA
Voxel size	70 μm
Filter	none
Exposure time	250 ms
Number of X-rays used to reconstruct a 3D image	3200

**Table 2:** Detailed results for the reference beams and the beams reinforced with CFRP.

Number of CFRP Reinforcement Layers		Specimen No.							
		1	2	3	4	5	6	7	8
0	F (kN)	24.55	17.67	18.61	18.09	17.75	18.6	17.67	17.54
	u (mm)	10.46	7.80	8.32	10.69	11.24	10.29	9.99	9.97
	EI (Nm^2^)	13,224	11,450	11,808	9615	11,309	11,072	9171	9354
1	F (kN)	17.68	26.46	26.64	25.21	24.55	25.32	23.97	25.50
	u (mm)	9.70	13.55	13.69	13.29	13.50	13.88	14.42	15.08
	EI (Nm^2^)	8989	12,733	11,250	10,420	9473	13,246	10,961	10,157
3	F (kN)	18.80	25.50	22.05	22.29	23.74	22.17	23.43	22.59
	u (mm)	11.37	16.22	10.83	12.47	12.81	11.82	12.27	12.92
	EI (Nm^2^)	10,042	9181	10,929	9784	9762	9314	9928	14,278
5	F (kN)	16.15	23.43	17.29	24.86	26.77	27.31	26.17	26.94
	u (mm)	8.46	10.86	7.68	11.84	9.92	9.58	9.44	9.01
	EI (Nm^2^)	8843	11,098	10,724	12,594	13,218	14,069	14,968	16,357

**Table 3:** Comparison of load-bearing capacities for the beams in the various series.

		Number of CFRP Reinforcement Layers			
		Without Reinforcement (for 7 Specimens)	1 Layer (for 7 Specimens)	3 Layers (for 7 Specimens)	5 Layers (for 8 Specimens)
Mean ultimate force for each beam series	[kN]	17.99	25.38	23.11	23.62
Standard deviation	[kN]	0.45	0.96	1.24	4.45
Coefficient of variation	[%]	2.52	3.76	5.35	18.84
Mean value of the bending moment for each beam series	[kNm]	2.04	2.88	2.62	2.68
Average value of the increase in the load-bearing capacity of the beams reinforced with CFRP in relation to the reference beams	[%]	-	41.07	28.46	31.29

**Table 4:** Failure mode of the tested beams.

Type of Beam	Failure Mode (Scheme 1 to 7 according to De la Rosa)	Dominant Course of Failure	Detachment of the Reinforcement
Reference beam From 0/1 to 0/8	**3** (tension)—7 times **6** (shear)—1 time	Fracture at the border of a growth ring (tangent)—delamination, tearing of the bottom fibers, destruction initiated and running through knots	not applicable
One layer of reinforcement from beam 1/1 to 1/8	**5** (shear)—6 times **6** (shear)—1 time **4** (shear + tension) + 2 compression—1 time	Shear of the cross-section-destructive longitudinal crack above the knot line, spreading from the center, fracture at the border of a growth ring (tangent)—delamination from the center towards the support or delamination going to the knot in the middle of the span	no
Three layers of reinforcement from beam 3/1 to 3/8	**6** (shear)—7 times **7** (no visible breakage)—1 time	Shear of the bottom part of the cross-section in the support zone, which runs to the center of the beam and passes through a knot (right next to the support), delamination of the beam	no
Five layers of reinforcement from beam 5/1 to 5/8	**5** (shear)—5 times **6** (shear)—3 times	Shear of the bottom part of the cross-section in the support zone, which runs horizontally to the center or which runs through the entire beam to the opposite top part of the cross-section, partial adhesive failure at the wood-glue interface, delamination of the element	no (6 times) a part next to a support (2 times)

## Data Availability

The data presented in this article are available within the article.

## Nomenclature and Symbols

f_m,g,k_	bending strength of the GL28h spruce wood
f_t,0,g,k_	tensile strength of the GL28h spruce wood
f_c,0,g,k_	compressive strength of the GL28h spruce wood
E_0,g,mean_	mean value of modulus of elasticity of the GL28h spruce wood
f_t,cf_	tensile strength of carbon fiber mats (MPa)
$E_{f}$	elastic modulus of the carbon fiber mats (GPa)
$\delta_{\mathrm{cf}}$	elongation at break of the carbon fiber mats (%)
$\delta_{\mathrm{er}}$	elongation at break of the epoxy resin (%)
E_er_	modulus of elasticity in flexural of the epoxy resin (GPa)
F_t,er_	tensile strength of the epoxy resin (MPa)
F	destructive force (kN)
l	span of the beam (m)
u	vertical displacement of the beam (mm)
${\mathrm{EI}}_{\mathrm{mean}\left(u=2–6\;\mathrm{mm}\right)}$	equivalent stiffness within the displacement range of 2–6 mm (Nm^2^)
CV	coefficient of variation (%)
